# Clinical efficacy of simplified intravoxel incoherent motion imaging using three b-values for differentiating high- and low-grade gliomas

**DOI:** 10.1371/journal.pone.0209796

**Published:** 2018-12-27

**Authors:** Takuya Hino, Osamu Togao, Akio Hiwatashi, Koji Yamashita, Kazufumi Kikuchi, Daichi Momosaka, Hiroshi Honda

**Affiliations:** 1 Department of Clinical Radiology, Graduate School of Medical Sciences, Kyushu University, Fukuoka, Japan; 2 Department of Molecular Imaging & Diagnosis, Graduate School of Medical Sciences, Kyushu University, Fukuoka, Japan; Medical University of Graz, AUSTRIA

## Abstract

In this study, we evaluated the efficacy of intravoxel incoherent motion (IVIM)-derived parameters calculated with three b-values in differentiating high-grade gliomas (HGGs) from low-grade gliomas (LGGs) by comparing those calculated with multiple b-values. Ten patients with LGG (ages 35.1±12.1 yrs; 4 males, 6 females) and 21 patients with HGG (ages 60.6±19.1 yrs; 10 males, 11 females) who underwent subsequent surgical resections were examined with both IVIM imaging and histopathological analysis. The IVIM diffusion-weighted imaging was conducted using a single-shot echo planar sequence with 13 b-factors (0, 10, 20, 30, 50, 80, 100, 200, 300, 400, 600, 800, and 1000 sec/mm^2^) at 3T. In the conventional IVIM analysis, the perfusion fraction (f) and true diffusion coefficient (D) were calculated by biexponential fitting model with 13 b-values. In the simplified method with the selected three b-values (0, 300, and 1000 sec/mm^2^), D simply corresponds to the slope of a straight line passing through two logarithmic signal intensities (SIs) at the b-values of 300 and 1000 s/mm^2^, and f corresponds to the difference between the intercept of this line and SI at the b-value of 0 sec/mm^2^. The maximum f (f-max) and minimum D (D-min) was measured in each tumor. The f-max values calculated with three b-values (12.8±5.9%) were significantly lower than those with 13 b-values (17.3±7.5%, p<0.0001), but a good correlation and agreement were observed between these sets of f-max values (r = 0.79, ICC = 0.87). In the IVIM imaging with both three and 13 b-values, the HGGs showed significantly higher f-max values compared to the LGGs (p<0.001, respectively). The D-min values calculated with three b-values (1.06±0.31 ×10^−3^ mm^2^/sec) was not different from those with 13 b-values (1.07±0.33 ×10^−3^ mm^2^/sec), and an excellent correlation and agreement were found between them (r = 0.99, ICC = 0.99). The simplified IVIM imaging using three b-values can efficiently differentiate HGGs and LGGs.

## Introduction

Glioma is a common tumor that arises from glial cells of the central nerve system [1, 2]. The differentiation between high-grade gliomas (HGGs; Grade III or IV) and low-grade gliomas (LGGs; Grade II) is essential for the determination of the therapeutic strategy [3, 4]. Gliomas are classified into four grades in accord with the World Health Organization (WHO) system, based on histopathological features: the presence or absence of cellular anaplasia and nuclear atypia, cell density, mitoses, microvascular proliferation, and necrosis [5, 6].

Magnetic resonance (MR) imaging can provide information associated with such histopathological features and has been used, for example, in preoperative diagnoses of benign and malignant brain tumors. Diffusion-weighted (DW) imaging plays an important role in the quantification of the tumor microenvironment and specifically in the assessment of tumors' cellular solid component [7]. In some studies, the apparent diffusion coefficient (ADC) measured by DW imaging showed high diagnostic performance in differentiating HGGs from LGGs, but a substantial overlap in ADCs between LGGs and HGGs was also observed [7–10]. Dynamic susceptibility contrast (DSC) perfusion-weighted (PW) imaging can provide information related to tumor vascularity, which is another important histopathological feature, and DSC-PW imaging has been used in the assessment of gliomas [11]. However, the use of DSC-PW imaging is frequently limited in patients with renal dysfunction or allergic factors, as well as in pediatric patients.

Intravoxel incoherent motion (IVIM) imaging is a concept and method for simultaneously measuring perfusion and diffusion, proposed by Le Bihan et al. [12, 13]. IVIM was defined as the microscopic translational motions of water molecules that occur in each image voxel in MR imaging [14]. Such motions include not only water diffusion but also microcirculations of blood in capillaries (perfusion) in *in vivo* tissues. The microvascular perfusion fraction (f), true diffusion coefficient (D) and perfusion-related coefficient (D*) are calculated with a bi-exponential fitting analysis, although accuracy and reproducibility of D* have remained unestablished possibly due to difficulties in fitting [15] or effect of cardiac cycle [16]. IVIM imaging has been reported to be useful for differentiating LGGs and HGGs [17–19].

The diffusion coefficient is measured as the negative slope in the plot of the logarithmic signal intensities obtained at multiple b-values (generally from 0 sec/mm^2^ to 1000 sec/mm^2^). DW images obtained with low b-values (e.g., <100 sec/mm^2^) are greatly influenced by the microcirculation in tissues, and thus higher b-values (e.g., 400 <b <1000 sec/mm^2^) have been used to estimate pure diffusion; in addition the characteristics of SI decay would change at b-values between 100 and 400 sec/mm^2^ [20, 21]. Previous IVIM studies typically used a more than ten b-values for fitting [17–19], but theoretically the D and f-values can be calculated using only three b-values [12]. Reducing the number of b-values can lead to a shortening of the scan time and improved clinical efficiency.

Sumi et al. reported that perfusion-related parameters that correspond to f-values calculated with three b-values were useful in differentiating the types of head and neck tumors [22]. More recently, diffusion-weighted imaging with three b-values were applied in the brain [23–25]. We conducted the present study to determine the efficacy and diagnostic performance of IVIM-derived parameters calculated with three b-values in differentiating HGGs from LGGs by comparing to those with multiple b-values.

## Materials and methods

This retrospective study was approved by our institutional review board of Kyushu University Hospital, and the requirement for patients' written informed consent was waived.

### Patients

IVIM imaging has been performed as a part of the routine protocol in preoperative examinations for patients with suspected brain tumor at our Departments of Clinical Radiology and Neurosurgery since February 2013. A total of 31 consecutive patients with diffuse glioma who underwent IVIM imaging during the period from February 2013 to April 2015 were included in the study. Diagnoses were made histopathologically by operation or biopsy in all 31 patients.

The LGG group was comprised of 10 patients (ages 35.1±12.1 yrs; 4 males, 6 females) and the HGG group was comprised of 21 patients (ages 60.6±19.1 yrs; 10 males, 11 females). The patients’ histological types of gliomas were as follows: 7 diffuse astrocytomas, 2 oligodendrogliomas, 1 oligoastroctyoma, 1 anaplastic astrocytoma, 1 anaplastic oligoastrocytoma, and 19 glioblastoma multiforme (GBM). All tumors were supratentorial. All patients underwent surgery within 2 weeks after the MR imaging.

### Intravoxel incoherent motion MRI

IVIM imaging was performed on a 3T clinical scanner (Achieva TX, Philips Healthcare, Best, The Netherlands) with an eight-channel head coil. DW MR imaging was performed in axial planes by using13 b-values (0, 10, 20, 30, 50, 80, 100, 200, 300, 400, 600, 800, and 1000 sec/mm^2^). Motion probing gradients were applied in three orthogonal directions. Fat suppression was performed by using the spectral inversion recovery method. The other imaging parameters were: repetition time = 2,500 msec; echo time = 70 msec; matrix = 128×126 (reconstructed to 256×256); slice thickness = 5 mm, field of view = 230×230 mm; number of slices = 11, sensitivity encoding factor = 1.5; scan time = 2 min 7 sec. The clinical protocol also included T1-weighted, T2-weighted, fluid attenuation inversion recovery (FLAIR), and post-contrast T1-weighted images.

### IVIM imaging analysis

The IVIM imaging analysis was performed using image analysis software (SYNAPSE VINCENT, Fujifilm Medical, Tokyo). We used the standard IVIM two-compartment diffusion model, with a capillary perfusion component and a nonvascular compartment. Signal decay was estimated by using the following biexponential equation.

$$\frac{SI}{SI0}=(1-f)•\exp\left(-bD\right)+f•exp\left(-bD*\right)$$

where D and D* are the true diffusion coefficient and the pseudodiffusion coefficient, respectively, SI is the signal intensity at a given b-value and SI_0_ is the signal intensity at b = 0 sec/mm^2^, and f is the fractional volume of the capillary perfusion. The SI_0_ was set to the measured signal at b = 0 sec/mm^2^. The SIs were fitted in two steps, first for b ≥300 sec/mm^2^ for the single parameter D, then for all b-values and all parameters while keeping D constant. The fitting was performed with the constraint on f (0 < f <1).

**Fig 1:**
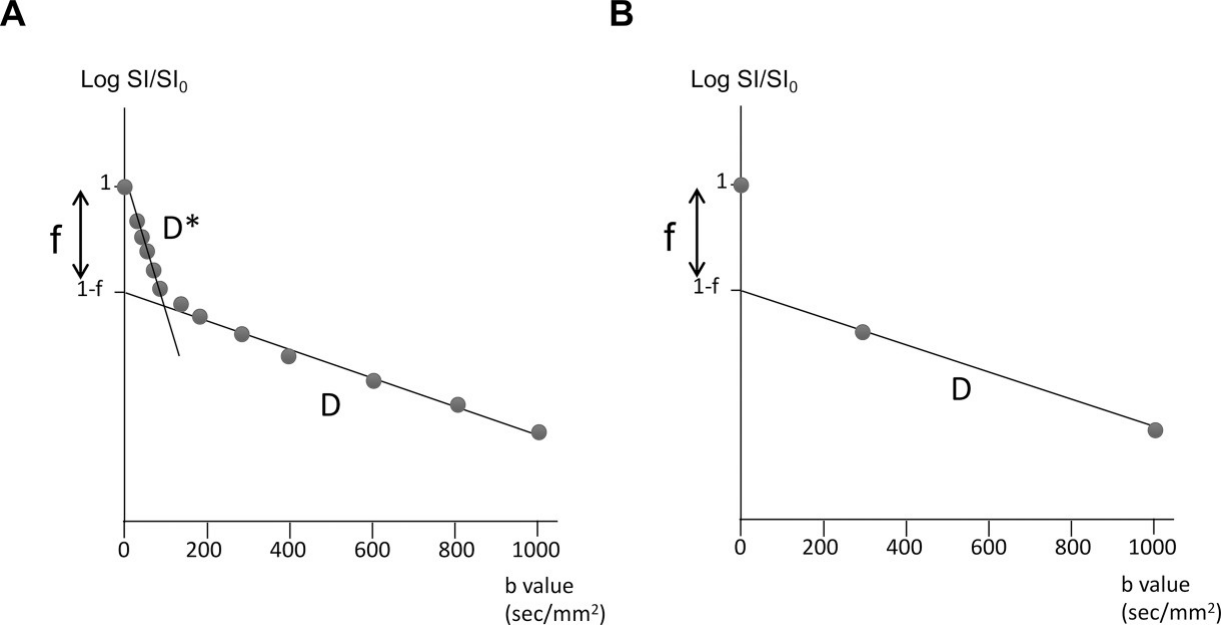
Fitting schemes. **(A)** In the fitting using 13 b-values, the SIs were biexponentially fitted in two steps, first for b ≥300 sec/mm^2^ for the single parameter D, then for all b-values and all parameters while keeping D constant. (**B)** In the fitting using three b-values (0, 300, 1000 sec/mm^2^), the D simply corresponds to the slope of the straight line passing through two logarithmic SIs at the b-values of 300 and 1000 sec/mm^2^, and f corresponds to the difference between the intercept of this line and S0.

In the analysis with the selected three b-values (0, 300, and 1000 sec/mm^2^), D simply corresponds to the slope of a straight line passing through two logarithmic SIs at the b-values of 300 and 1000 s/mm^2^, and f corresponds to the difference between the intercept of this line and SI_0_ (**Fig 1**). The hot-spot method was used to measure IVIM parameters as used in previous studies [19, 26]. Regions-of-interest (ROIs) were carefully placed in three circular regions of interest (ROIs, approx. 0.26 cm^2^, 32 pixels) in the solid component of the tumor to include the area with the minimum D (D-min) or maximum f-value (f-max), and the best effort was given to avoid cystic, necrotic, and hemorrhagic components of the tumor with reference to conventional MR images. The three ROIs were placed by a radiologist (5 years' experience), and another radiologist (17 years' experience) approved them. The ROIs were placed on the maps measured with the 13 b-values, and the same ROIs were used for the maps measured with the three b-values. Measurements in the three ROIs were averaged to represent a tumor.

### Histopathological evaluation

The histopathological diagnosis was determined with resected specimens according to the WHO criteria (5).

### Statistical analysis

All values are expressed as the mean±standard deviation. The f-max and D-min measured with the three b-values were compared with those obtained with the 13 b-values by paired t-test. Correlations and agreements between values were assessed with Pearson's correlation and intra-class correlation coefficient (ICC), respectively. The f-max and D-min were compared between the LGG and HGG groups by unpaired t-test. We used a receiver operating characteristic (ROC) analysis to evaluate the diagnostic accuracy of age, D, f and combination of D and f in differentiating LGGs from HGGs. We considered area under the curve (AUC) values < 0.7, 0.7–0.9, and > 0.9 to indicate low, medium, and high diagnostic performance, respectively. Statistical analyses were performed with a commercially available software package (SPSS, IBM 19, Armonk, NY; Prism 5.0, GraphPad Software, San Diego, CA; and MedCalc v. 13.1.2.0, Broekstraat, Mariakerke, Belgium). P-values <0.05 were considered significant.

## Result

### Analysis of IVIM parameters

**Fig 2:**
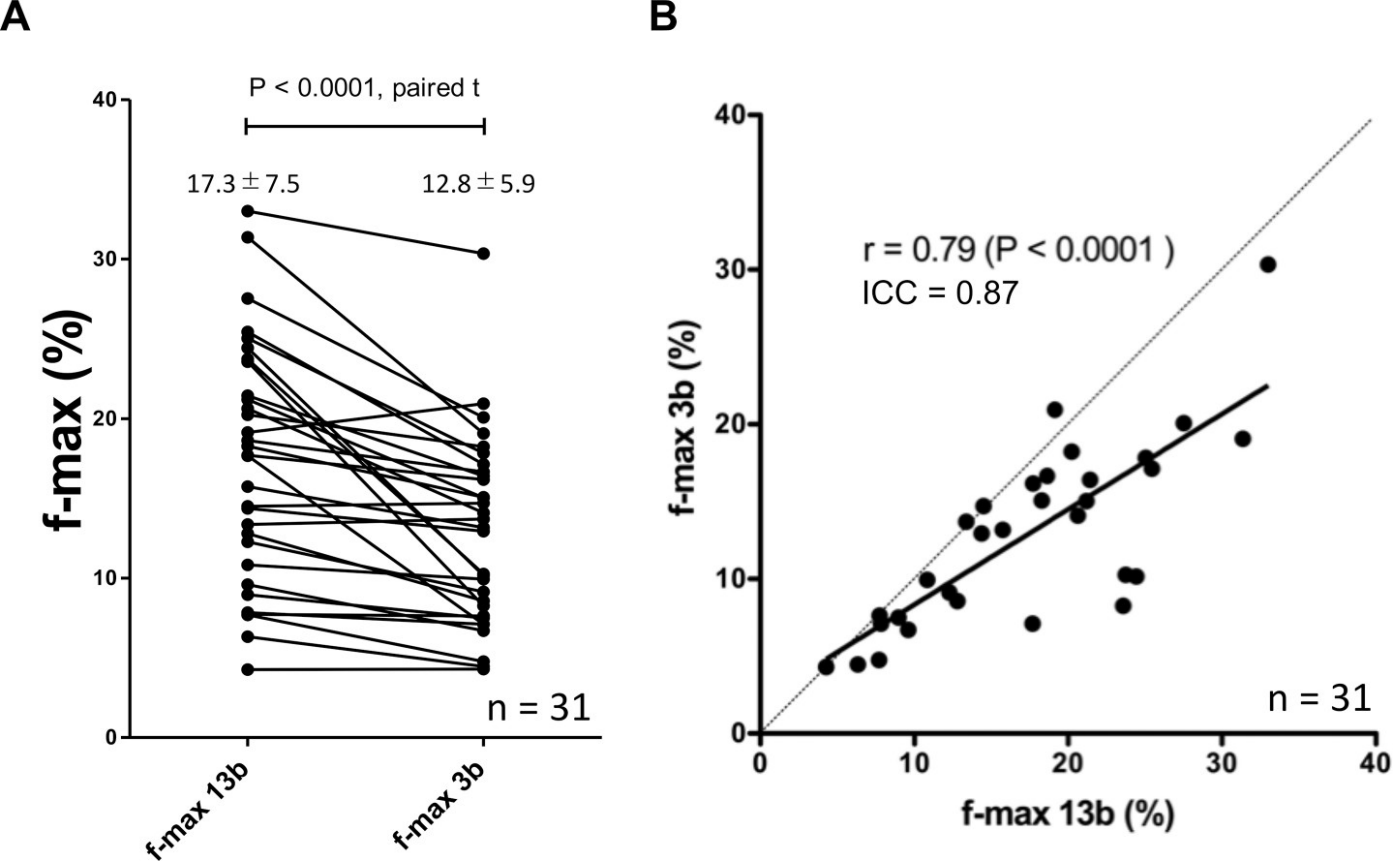
Comparison and correlation of f-max values. The f-max values calculated with the three b-values was significantly lower than those calculated with the 13 b-values (**A**), but a good correlation (r = 0.79) and agreement (ICC = 0.87) were observed between them (**B**).

**Fig 3:**
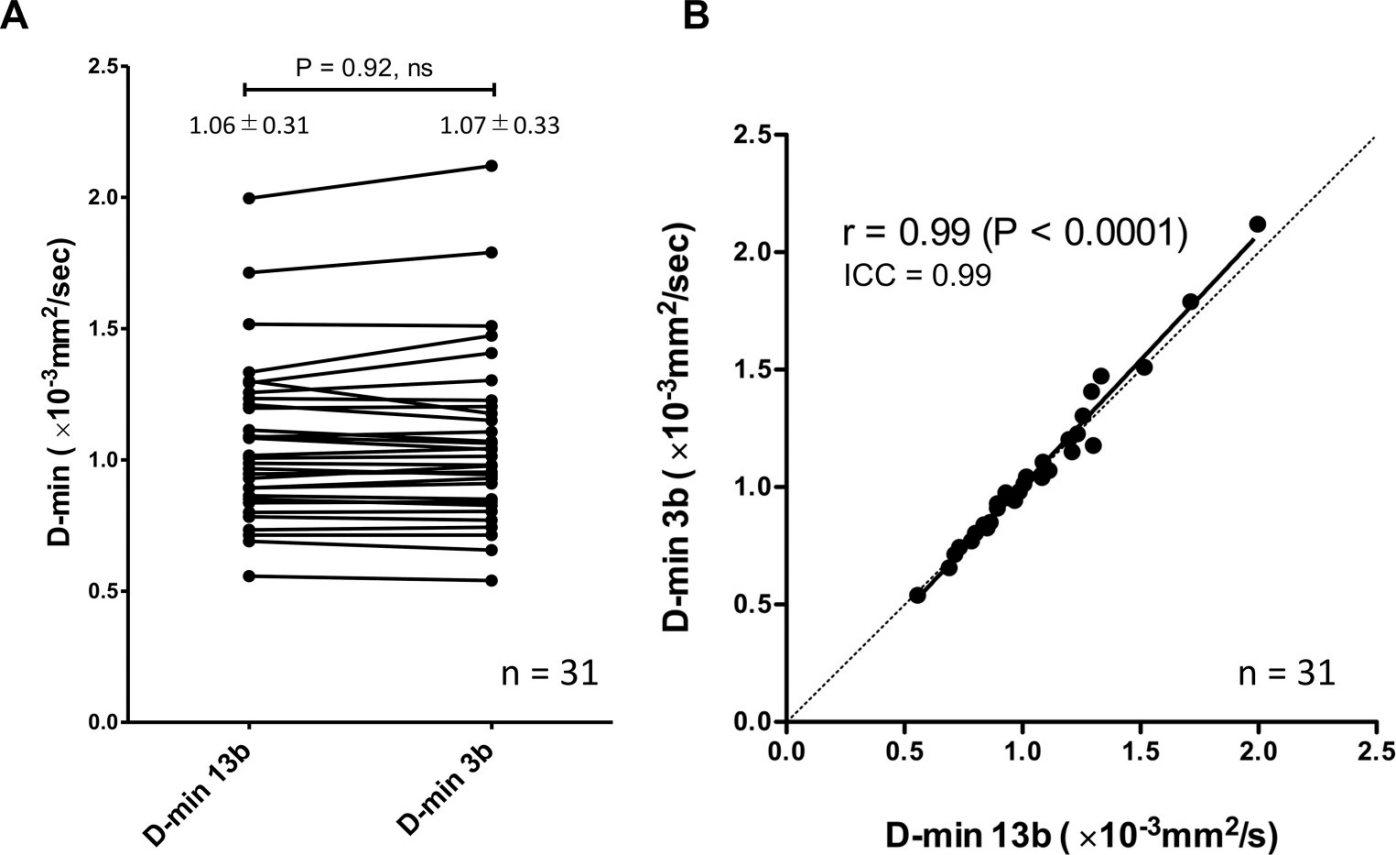
Comparison and correlation of D-min values. The D-min values calculated with the three b-values were not significantly different from those calculated with the 13 b-values (**A**), and an excellent correlation (r = 0.99) and agreement (ICC = 0.99) were found between them (**B**).

The f-max values calculated with the three b-values (12.8±5.9%) were significantly lower than those calculated with the 13 b-values (17.3±7.5%, p<0.0001), but a good correlation (r = 0.79) and agreement (ICC = 0.87) were observed between these two sets of values (**Fig 2**). The D-min value calculated with the three b-values (1.06±0.31 ×10^−3^ mm^2^/sec) was not significantly different from those obtained with the 13 b-values (1.07±0.33 ×10^−3^ mm^2^/sec), and an excellent correlation (r = 0.99) and agreement (ICC = 0.99) were found between them (**Fig 3**).

### Differentiation between LGGs and HGGs

**Fig 4:**
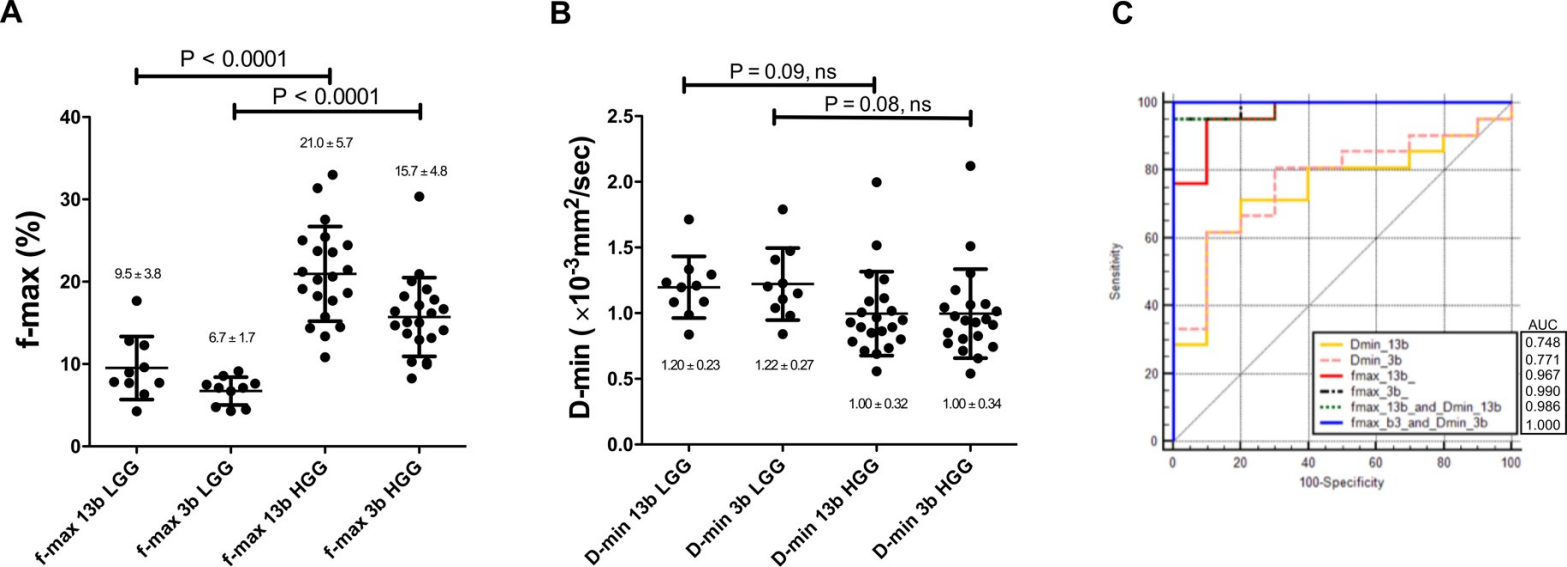
f-max and D-min values in HGGs and LGGs. The f-max value obtained with both the 13 and the three b-values was significantly higher in the HGG group than in the LGG group (**A**). The HGG group tended to show lower D-min compared to the LGG group at both b-value settings, but no significant difference was found between the two groups (**B**). The horizontal line represents the mean value, and the error bars represent the standard deviation. The diagnostic performance of each parameter in differentiating HGG from LGG (**C**). The ROC analysis for discriminating HGG from LGG demonstrated that the f-max obtained with both 13 b-values and 3 b-values shows the excellent diagnostic performance. The D-min obtained with both 13 b-values and 3 b-values shows moderate diagnostic performance. The combination of f-max and D-min increased the AUC for both sets of b-values.

**Fig 5:**
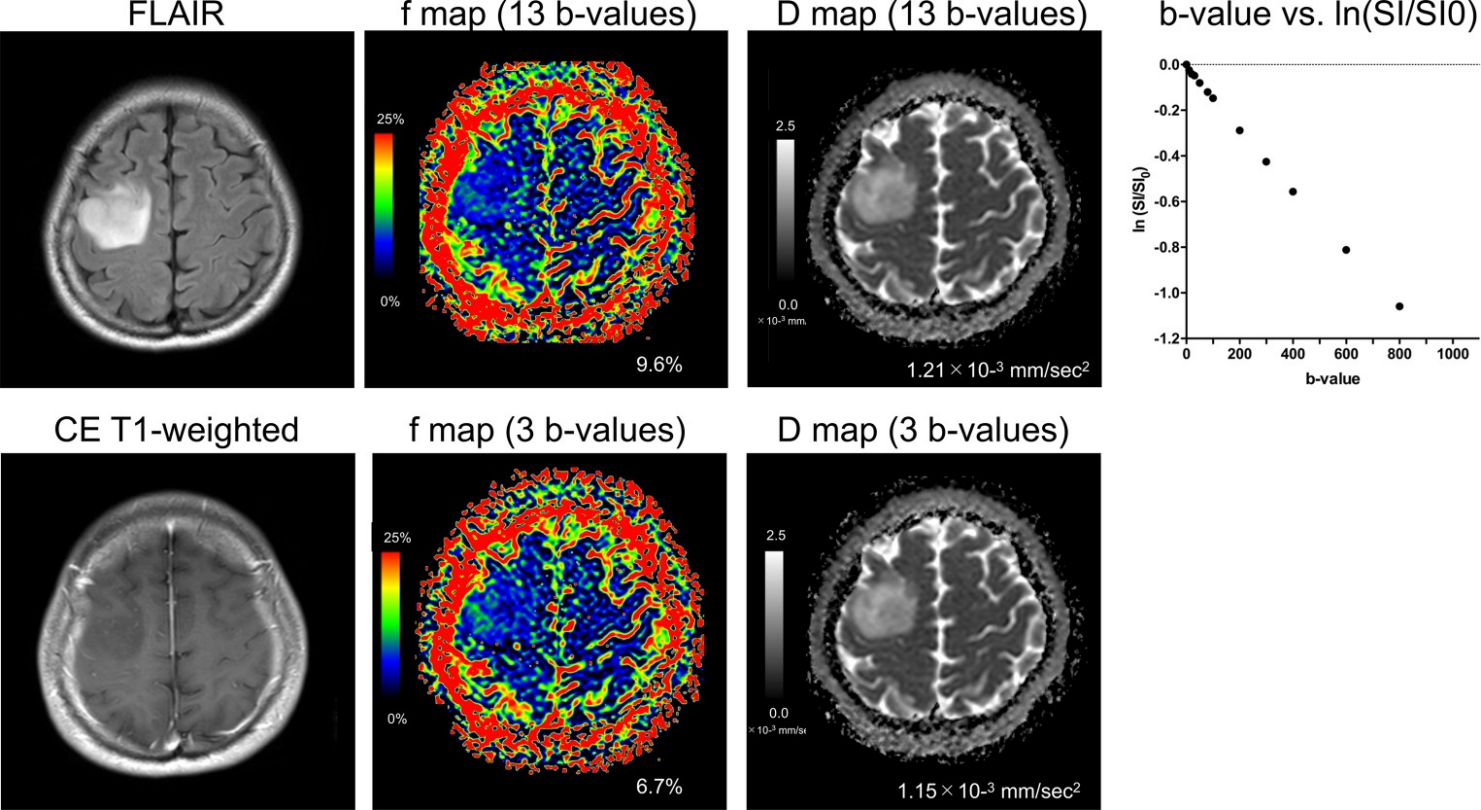
A 38-year-old-male with diffuse astrocytoma (low-grade glioma). The lesion shows low f-values and high D values on both b-value settings. The f-max value measured with the three b-values was slightly smaller than those obtained with the 13 b-values, whereas the D-min values were almost identical in both b-value settings. The representative plot of ln(SI/S0) against b-value in the dashed circle shows the linear slope of signal decay for b-values near 0 sec/mm^2^ as well as in b-values > 300 sec/mm^2^.

**Fig 6:**
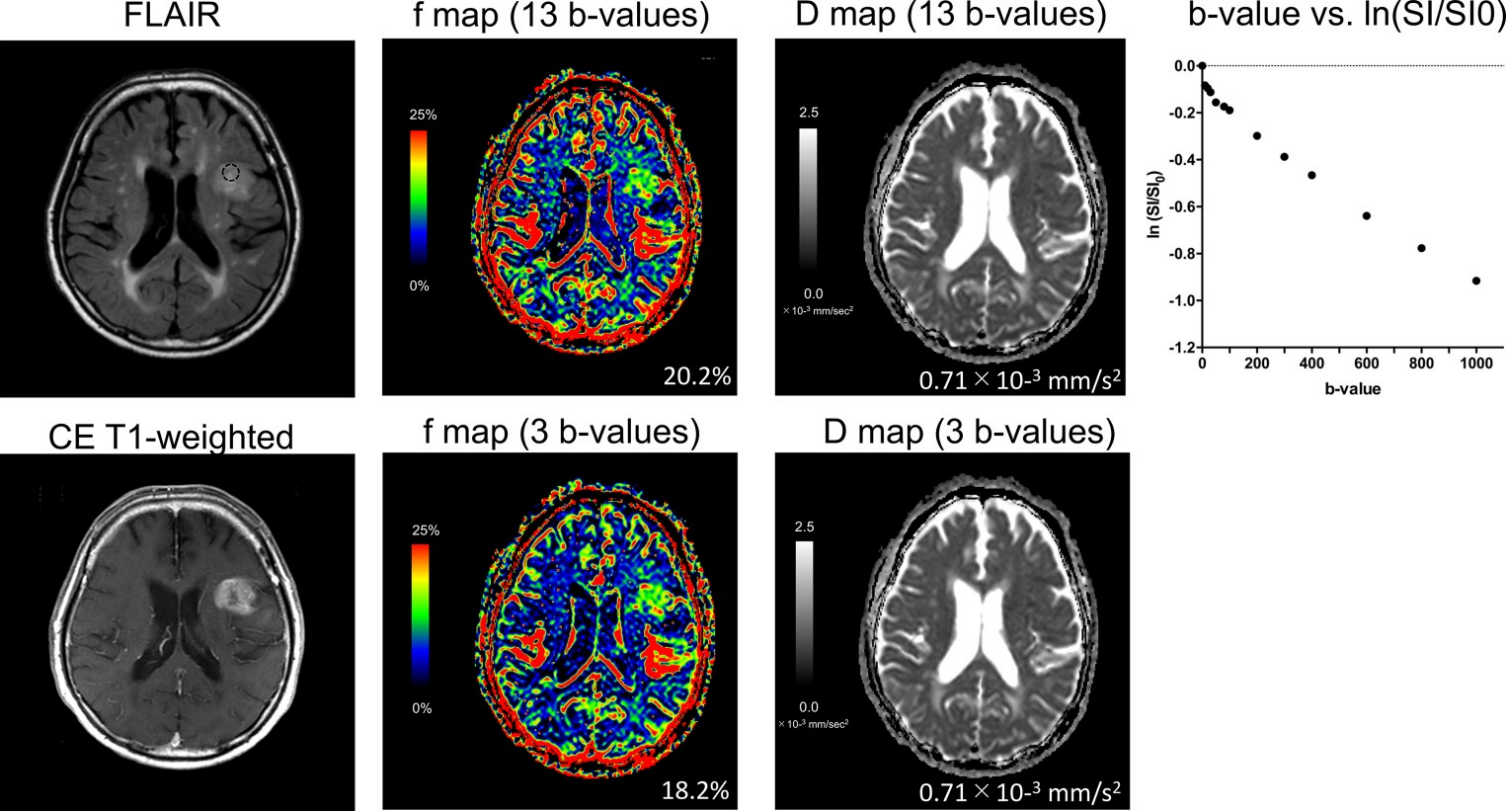
A 78-year-old-male with glioblastoma (high-grade glioma). The lesion shows higher f-max values and lower D-min values on both b-value settings compared to the low-grade glioma in Fig 5. The f-max value measured with the three b-values was slightly smaller than those measured with the 13 b-values, whereas the D-min values were identical in both b-value settings. The representative plot of ln(SI/S0) against b-value in the dashed circle shows a steeper slope of signal decay for b-values near 0 sec/mm^2^ than in b-values > 300 sec/mm^2^.

The f-max value obtained with both the 13 and the three b-values was significantly higher in the HGG group (13 b-values, 21.0±5.7%; three b-values, 15.7±4.8%) compared to the LGG group (13 b-values, 9.5±3.8%; three b-values, 6.7±1.7%) (p<0.0001, respectively, **Fig 4A**). The HGG group (13 b-values, 1.00±0.32 ×10^−3^ mm^2^/sec; three b-values, 1.00±0.34 ×10^−3^ mm^2^/sec) tended to show lower D-min values compared to the LGG group (13 b-values, 1.20±0.23 ×10^−3^ mm^2^/sec; three b-values, 1.22±0.27 ×10^−3^ mm^2^/sec) at both b-value settings, but no significant difference was found between the two groups (**Fig 4B**). **Figs 5 and 6** shows representative cases of LGG and HGG, respectively. The HGG shows a higher f-value and lower D-value compared to the LGG. In both cases, the f-values obtained with the three b-values were slightly smaller than those obtained with the 13 b-values, whereas the D-values were almost identical in both b-value settings. The diagnostic performance of each parameter in differentiating HGG from LGG is shown in Fig 4C. The results of the ROC analysis for discriminating HGG from LGG demonstrated that the f-max obtained with both 13 b-values and 3 b-values showed the excellent diagnostic performance with AUC values of 0.967 and 0.990, respectively. The D-min obtained with both 13 b-values and 3 b-values showed moderate diagnostic performance with AUC values of 0.748 and 0.771, respectively. The combination of f-max and D-min increased the AUC for both sets of b-values. The patient’s age showed moderate diagnostic performance with AUC value of 0.864 (not shown in the graph).

## Discussion

Our present findings elucidated that the f-max calculated with the three b-values showed good agreement with the f-max calculated with the 13 b-values, although the f-max values obtained with the three b-values were smaller. This good agreement suggests that the possible errors of f-values calculated with the minimum number of b-values is within the acceptable range and that this value is reliable enough for the clinical use. However, the f-max values obtained with the three b-values were significantly lower than those obtained with the 13 b-values. Conklin et al. reported that reducing the number of b-values in the estimation of f-value resulted in the reduced accuracy compared with full biexponential fitting [25]. However, their study also showed that the evaluation with three b-values still effectively differentiated HGGs from LGGs. In their study, the f-values obtained with three b-values (0, 300, 900 sec/mm^2^) were lower than those with full biexponential fitting. Their results are consistent with ours. The underestimation of the f-value in the simplified method might be due to the influence of remaining perfusion effects on the SI observed at the selected b-value. In their study, the increase in number of b-values over 300 sec/mm^2^ resulted in the disappearance of significant difference. This fact indicated that the underestimation of the f-value due to remaining perfusion effect might be corrected by using higher b-values than 300 sec/mm^2^.

Regarding diagnostic performance of IVIM parameters in differentiating HGGs from LGG, the f-max obtained with both 13 and three b-values showed better AUCs (0.967 and 0.990, respectively) compared with those of the D-min (0.748, 0.771, respectively). In addition, the combination of the f-max and D-min resulted in improved diagnostic performance for both b-value sets. Togao et al. showed using the full biexponential fitting that the f-value provided the highest diagnostic performance in discriminating HGGs from LGGs, with AUC value of 0.95, while D showed moderate diagnostic performance, with AUC values of 0.78 [19]. The results in the present study are almost equivalent to these results, assuring the robustness and accuracy of the present study. Cao et al. evaluated the feasibility of a simplified method based on DW imaging acquired with three b-values, and simplified perfusion fraction calculated from two ADC values measured with b-value sets of 0–200 and 200–1000 sec/mm^2^ [23]. Among all imaging parameters, simplified perfusion fraction achieved the highest AUC value of 0.942, followed by f (0.896), and D* (0.891), whereas the AUC value of D-value (0.732) was relatively low. Since they did not evaluate f-values obtained with three b-values, we could not directly compare our results with theirs. However, it seems that perfusion parameters obtained with three b-values have substantial clinical utility in differentiating HGGs from LGGs.

Simplified IVIM techniques have been used to assess head and neck tumors [22], liver cirrhosis [27], hepatocellular carcinomas [28], and gliomas [25]. The b-values used to extract the true diffusion component in those studies ranged from 200 to 500 sec/mm^2^. The cutoff b-value of 300 sec/mm^2^ used in our study is within this range. Use of different cutoff b-values can cause bias in the estimation. However, no optimal cutoff b-value has not been established for brain tumors yet. Meeus et al. investigated the reliability of parameters D and f and their dependence on b-value distributions with the three b-value protocol, and found that the effect of bias was higher for the low-perfused organ and the optimal b-value for the brain was 500 sec/mm^2^ [29]. On the contrary, the effect of bias was considerably lower for the high-perfused models and the optimal b-value for kidney and liver was 300 and 200 sec/mm^2^, respectively [29]. Since perfusion of gliomas increased with their histologic grade, optimal cutoff b-value would change depending on the histological grade. Considering increased perfusion in HGGs, the cutoff b-value of 300 sec/mm^2^ used in our study may be reasonable. Further studies are necessary to identify the optimal b-value to exclude the perfusion effect. Despite this discrepancy, the f-value calculated with the three b- values was able to differentiate LGGs and HGGs. This indicates that the simplified IVIM imaging described herein is effective for clinical use.

In contrast, the D-min values calculated with the three b-values showed excellent agreement with those obtained with the 13 b-values. This could be due to the locational differences between D-min and f-max. The D-min values could be less influenced by the perfusion effect since the area with a high f-max did not always correspond to that with a low D-min. The D-min tended to be lower in the HGGs than in the LGGs, but the difference did not reach significance. This indicates that the f-value could have a higher diagnostic performance than the D-value in differentiating HGGs from LGGs, which is consistent with results of previous studies [17–19].

The simplified three-point b-value sampling can greatly contribute to the shortening of the total acquisition time compared to conventional multiple (e.g., >10 points) b-value sampling. In the present study, the total acquisition time in all of the 13 b-values' acquisitions was 126 sec. This could be shortened to 29 sec if we reduced the number of b-value to three. It is of clinical benefit to obtain perfusion-related information with simplified IVIM imaging by simply adding one b-value to conventional DW imaging, which typically use a pair of b-values (e.g., 0 and 1000 sec/mm^2^).

The patient’s age was a significant factor in the discriminating HGGs from LGGs. It is well known that higher grade glioma high-grade gliomas (HGGs) tend to occur at increasing rates with increasing age. For example, the mean age of presentation of glioblastoma is 61.3 ± 14.0 years [30], whereas that of low grade glioma is 41.4 ± 15.6 years [31]. In the ROC analysis, the patient’s age showed high AUC value of 0.864, indicating that patient’s age could be a useful parameter to differentiate HGGs from LGGs.

This study has limitations. First, the number of patients was relatively small (especially that of the LGG group; n = 10). This could be the reason why we did not observe significant differences in the comparison of D-min values between the LGG and HGG groups. Second, we did not evaluate b-values other than 300 sec/mm^2^ for the measurements, since many analyses including other b-values could complicate this study. Third, another IVIM-derived parameter, D*, was not assessed in this study because accurate D* could not be calculated with three b-values. In addition, previous studies suggested that D* was less reproducible [15] and could be strongly affected by the cardiac cycle [16] as mentioned above. Finally, we used the hot-spot method to measure IVIM parameters rather than whole-tumor histogram analysis. However, it has been recently reported that whole-volume histogram analysis did not yield better results than the hot-spot method and took longer time [32].

## Conclusions

The f-max values calculated with three b-values showed good agreement with those obtained using 13 b-values, although the former were lower values. The D-min values calculated with three b-values showed excellent agreement with those calculated with 13 b-values. Both f- and D-values obtained with the two b-value settings were useful in differentiating HGGs from LGGs. The simplified IVIM imaging with three b-values described herein can be clinically efficient in the differentiation of HGGs and LGGs.
